# MiR499-5P loaded MSC derived exosomes affect oxidative stress and inflammatory response after spinal cord injury by targeting genes

**DOI:** 10.1186/s13018-025-06500-w

**Published:** 2025-12-10

**Authors:** Likai Pang, Le Qi, Hang Wang, Tao Zhu, Yi Wang

**Affiliations:** 1 Department of Neurosurgery, Tianjin Medical University General Hospital, Tianjin, China; 2 Key Laboratory of Post-Trauma Neuro-Repair and Regeneration in Central Nervous System, Ministry of Education & Key Laboratory of Injuries, Variations and Regeneration of Nervous System in Tianjin, Tianjin Neurological Institute, Tianjin, China; 3 Department of Trauma Surgery, Tianjin First Central Hospital, Tianjin, China; 4 Department of Orthopaedics, The First Affiliated Hospital of Harbin Medical University, Harbin, China

**Keywords:** Spinal cord injury, miRNA, MSC-EXO-miR499-5P, MSC-EXO

## Abstract

Spinal cord injury (SCI) is a severe and prevalent disorder of the central nervous system that leads to sensory and motor dysfunction, with approximately 700,000 new cases reported annually. Recently, miRNA-mRNA regulatory networks have garnered significant attention from researchers. Preliminary evidence from animal models and bioinformatic analyses suggests that altered miRNA expression affects critical pathophysiological processes in SCI. Therefore, the miRNA-target gene interaction network holds promise as a potential therapeutic avenue to promote neural recovery post-SCI. In this study, we demonstrated for the first time that MSC-EXO-miR499-5P can protect neurons and promote the recovery of motor function in SCI mice. First, we selected the GSE2599 dataset through bioinformatic analysis and identified differentially expressed genes (DEGs) using the limma package. Subsequently, we focused on the upregulated DEGs enriched in the Kyoto Encyclopedia of Genes and Genomes (KEGG) pathways related to oxidative stress, inflammatory responses, and apoptosis. Following the comparative analysis, miR499-5P was selected for further in vitro and in vivo investigations. Subsequent evaluations demonstrated that MSC-EXO-miR499-5P promoted cell growth, alleviated post-injury oxidative stress and inflammation, and contributed to the repair of the spinal cord microenvironment while enhancing functional recovery in SCI mice. In summary, our findings suggest that MSC-EXO containing miR499-5P represents a novel therapeutic approach for the treatment of SCI.

**Supplementary Information:**

The online version contains supplementary material available at 10.1186/s13018-025-06500-w.

## Introduction

Spinal cord injury (SCI) is a severe and prevalent disorder of the central nervous system, resulting in sensory and motor dysfunction, with approximately 700,000 new cases reported annually [1]. The etiology of SCI is multifactorial and includes high-impact trauma, infections, tumors, degenerative spinal diseases, ischemia–reperfusion injury, and vascular abnormalities [2–4]. The lifetime healthcare costs for patients with SCI can reach up to $4.6 million, imposing significant psychological and economic burdens on patients and their families [5–7]. Therefore, the development of effective and feasible therapeutic strategies is imperative. However, due to the complex pathophysiology of SCI and the limited regenerative capacity of neurons, current treatments remain challenging [8–10].

Pathologically, SCI progresses through primary and secondary injury phases. Primary injury refers to the initial mechanical damage (e.g., contusion or transection) caused by compression or traction at the moment of trauma [11–15]. Secondary injury involves a cascade of pathophysiological events—including neuronal death, ischemia–reperfusion, edema, neuroinflammation, and oxidative stress—mediated by interactions between the immune, nervous, and circulatory systems. These processes exacerbate spinal cord dysfunction and hinder recovery [16–18]. Consequently, therapeutic efforts should prioritize modulating the injury microenvironment to mitigate secondary damage.

Although glucocorticoids are widely used in acute SCI to suppress inflammation and oxidative stress [19, 20], they lack efficacy in restoring sensory and motor function during chronic phases [21, 22]. This underscores the need to elucidate post-SCI mechanisms and identify novel therapeutic targets [23–28].

Recent studies highlight the regulatory roles of miRNA-mRNA networks in SCI [29–34]. miRNAs are endogenous, single-stranded non-coding RNAs (~ 12–23 nucleotides) that bind complementary mRNA targets to post-transcriptionally regulate gene expression via translation inhibition or mRNA degradation [19, 35–37]. They are ubiquitously expressed in the brain, spinal cord, serum, urine, and cerebrospinal fluid [38–40], and modulate critical processes such as cell proliferation, differentiation, apoptosis, metabolism, and immune responses [41–44]. Notably, miRNAs regulate ~ 90% of human genes, influencing development and tissue homeostasis [45, 46]. Animal models and bioinformatic analyses suggest that dysregulated miRNAs contribute to SCI pathophysiology [47–50], making miRNA-target networks promising therapeutic avenues for neural repair.

In this study, we report for the first time that miR499-5P loaded MSC derived Exosomes (MSC-EXO-miR499-5P) promotes neuroprotection and the recovery of functional in mice with SCI. Using the GSE2599 dataset and the limma package, we identified differentially expressed genes (DEGs) and selected oxidative stress-, inflammation-, and apoptosis-related pathways from the upregulated DEGs via Kyoto Encyclopedia of Genes and Genomes (KEGG) enrichment. MiR499-5P was selected for further in vitro and in vivo validation. Our results demonstrated that MSC-EXO-miR499-5P enhanced cell viability, attenuated post-injury oxidative stress and inflammation, and ultimately improved the spinal cord microenvironment in SCI mice. Collectively, MSC-EXO that deliver miR499-5P may represent a novel therapeutic strategy for SCI.

## Materials and methods

### Data acquisition and preprocessing

We downloaded the GSE2599 dataset from the Gene Expression Omnibus (GEO) database (https://www.ncbi.nlm.nih.gov/geo/summary/), which contains the gene expression profiles of three spinal cord injury samples and three uninjured control samples. This dataset was based on the GPL85 [RG_U34A] Affymetrix Rat Genome U34 Array, which contains 8799 probes. To remove low-quality probes, we eliminated the probes that matched multiple genes and averaged the probes that matched the same gene. Finally, the expression matrix was normalized using the normalize Between Arrays function in the limma package.

### Identification and functional annotation of differentially expressed genes

To identify DEGs between SCI and normal controls, we used the limma package to calculate differences in gene expression between groups, with *p*-values corrected using the FDR method. Genes with |logFC|> 1 and adjusted *P* value < 0.05 were defined as DEGs. We then uploaded up- and down-regulated DEGs separately to the DAVID database (https://david.ncifcrf.gov/) for Gene Ontology (GO) annotation and KEGG pathway analysis of their associated biological functions, with a threshold of *p* < 0.05.

### Prediction of miRNA-target gene interactions

The miRTarBase database (http://mirtarbase.mbc.nctu.edu.tw/php/index.php) is a specialized resource that collects experimentally validated miRNA-mRNA target interactions (MTIs). To predict miRNA-target gene interactions, we searched miRTarBase for miRNAs that target oxidative stress-, inflammation-, and apoptosis-related genes.

### Cell culture and animals

Rat mesenchymal stem cells (MSC), 293 T cells and PC12 cells were purchased from Shanghai Meixuan Biological Science and Technology Co. Ltd. (Shanghai, China). All three cell types were cultured in high-glucose DMEM medium containing 10% exosome-free fetal bovine serum, 100U/ml penicillin and 100 ng/ml streptomycin, and maintained at 37 °C in a 5% CO2 incubator.

All animal studies were approved by the Tianjin Medical University General Hospital. Male C57BL/6j mice (6–8 weeks old, SPF grade) were provided by Spebio Biotechnology Company (Beijing, China) and housed at the Tianjin Medical University General Hospital. During the rearing period, the mice were provided with adequate water and food under well-ventilated conditions with appropriate temperature and humidity. The bedding was changed regularly under a 12 h light/dark cycle.

### Exosomes isolation, characterization and engineered exosome construction

The MSC culture medium was collected and centrifuged to remove cells and debris. The supernatant was transferred to new tubes and mixed with an appropriate volume of the exosome extraction reagent. After overnight incubation at 4 °C, exosomes were pelleted by centrifugation at 10,000 g for 1 h at 2–8 °C. The supernatant was discarded, and the exosome pellet was resuspended and stored at the appropriate temperature. For exosome characterization, nanoparticle tracking analysis (NTA) was used to measure particle size, transmission electron microscopy (TEM) was used to determine morphology, and western blotting was used to confirm successful exosome isolation.

Chemically synthesized miRNA mimics were diluted to 20 μM stock solutions with ddH_2_O. The mimics were mixed 1:1 with Namipo transfection reagent, incubated at room temperature for 10 min, added to MSC-EXO, and incubated at 37 °C for 5–6 h. After incubation, exosomes were re-isolated following the exosome extraction protocol to remove free miRNA, yielding engineered MSC-EXO-miRNA.

### Dual-luciferase reporter assays

Wild-type and mutant mRNA-3'UTR dual-luciferase reporter vectors were constructed and cotransfected with chemically synthesized miR499-5P mimic (Hereinafter referred to as miR499-5P) or miR499-5P negative control (miR499-5P-NC) into 293 T cells. After an appropriate incubation period, dual-luciferase activity was measured using a Dual-Luciferase Reporter Assay Kit (Beyotime, RG027).

### LPS-induced oxidative stress and inflammatory injury in PC12 Cells

PC12 cells were seeded in 6-well plates and cultured to approximately 70–80% confluence. To establish an in vitro inflammatory injury model, the cells were stimulated with lipopolysaccharide (LPS, *Escherichia coli* serotype O111:B4; Sigma-Aldrich, St. Louis, MO, USA) at a final concentration of 1 µg/mL for 48 h. MSC-EXO-miR499-5P or MSC-EXO-miR499-5P-NC was added simultaneously with LPS.

### Quantitative polymerase chain reaction assays

Total RNA was isolated from cells using TRIzol reagent (Sigma, China). 2 ug of RNA were reverse-transcribed into cDNA using the cDNA reverse transcription kit (Thermo Fisher Scientific, USA). Quantitative polymerase chain reaction (qPCR) was carried out with a SYBR Green PCR kit (Roche, Shanghai, China) on a CFX96 real-time PCR detection system (Bio-Rad Laboratories, Inc.). The amplification protocol consisted of pre-denaturation at 94 °C for 2 min, followed by 35 cycles of denaturation at 94 °C for 10 s, annealing at 60 °C for 15 s, and extension at 72 °C for 30 s, with a final extension at 72 °C for 5 min. Primers were synthesized by Sangon Biotech (China), and the sequences are listed in Table S1. GAPDH was used as the reference gene. The 2^−ΔΔCt^ method was used for analysis.

### SCI model construction and animal treatment

The mice were weighed preoperatively and anesthetized by intraperitoneal injection of 1% sodium pentobarbital solution (50–70 mg/kg). A laminectomy was performed to expose the T9–T11 spinal cord. Using the exposed dorsal spinal vessels as the center point, SCI was induced using an RWD spinal cord impactor by dropping a 5 g weight from a height of 10 cm. Successful modeling was confirmed by tail spasms and hind limb or body retraction movements. After complete hemostasis, the muscle fascia and skin were sutured layer by layer. Postoperatively, the mice received penicillin injections and nursing care for seven days to prevent infection. Manual bladder massage was performed three times daily until reflexive bladder control returned.

In this study, the details of MSC-EXO-miR499-5P administration were as follows: each mouse received a daily injection of 50 μL of MSC-EXO-miR499-5Psolution for seven consecutive days (a total of 350 μL). The injection site was located near the dorsal wound, approximately 2 mm from the center of the spinal cord injury. The injection depth was in the paraspinal region to ensure that the exosomes were evenly distributed around the injured area while avoiding direct entry into the intradural space. A 26-gauge needle (12 mm in length, 0.45 mm in diameter) was used for administration to allow slow and even delivery to the target region. The infusion rate was maintained at 50 μL/min to prevent local edema or tissue compression caused by rapid injection.

To provide additional details on the particle concentration, total particle number per injection, and protein content, we referred to the dose-dependency comparison of MSC-EXO administration routes suggested by Kostennikov et al. [51] and Shulman et al. [52]. Specifically, the particle concentration of MSC-EXO in each injection (measured by nanoparticle tracking analysis, NTA) was 2.5 × 10^11^ particles/mL, with a total of 1.25 × 10⁹ particles per injection and a protein content of 5 μg per injection. Additionally, we adjusted our administration protocol based on these studies to ensure a more accurate dose-dependent comparison in our evaluation.

BMS (Basso Mouse Scale) scoring was performed on postoperative days 1, 2, 3, 4, and 7 to assess motor function. The mice were sacrificed on day eight for spinal cord tissue collection.

### Western blotting analysis

RIPA lysis buffer (Beyotime Biotechnology, Shanghai, China) was added to cell or tissue samples. After complete lysis, samples were centrifuged at 12,000 rpm for 5 min to collect supernatant. Protein concentration was determined using BCA Protein Assay Kit (Beyotime Biotechnology, Shanghai, China). Protein samples were separated by SDS-PAGE and transferred to PVDF membranes. After blocking with 5% BSA for 2 h at room temperature and washing 3 times with TBST (5 min each), membranes were incubated with primary antibodies (Details of primary antibodies are shown in Table S2) overnight at 4 °C. After TBST washing (3 × 5 min), membranes were incubated with secondary IgG antibodies (1:1000, A-21206, Thermo Fisher, USA) for 2 h at room temperature. Following 5 × 15 min TBST washes, signals were detected using ECL reagent (2 min exposure) and X-ray film development.

### Flow cytometry

The culture medium was collected, and the cells were trypsinized without EDTA. The cell suspensions were centrifuged at 1,000 rpm for 5 min. After removing the supernatant, the cells were washed with ice-cold PBS and centrifuged. The cells were washed with 1 × binding buffer and adjusted to 1–5 × 10^6/ml. A 100 μl cell suspension was added to the flow tubes with 5 μL Annexin V/FITC, mixed gently, and incubated at room temperature for 5 min in the dark. 10 μL propidium iodide (PI) solution and 400 μL PBS were added, followed by immediate flow cytometry analysis.

### Statistical analysis

GraphPad Prism (v9.5.0) software was used for all statistical analysis. Three samples (n = 3) were included in each group, and results were expressed as mean plus standard deviation. Differences between multiple experimental groups were examined by one-way analysis of variance (ANOVA) and Tukey's multiple comparison test. “ns” means non-significant differences, *P* < 0.05 was considered to indicate statistical significance.

## Results

### Data processing and DEG identification

**Fig. 1:**
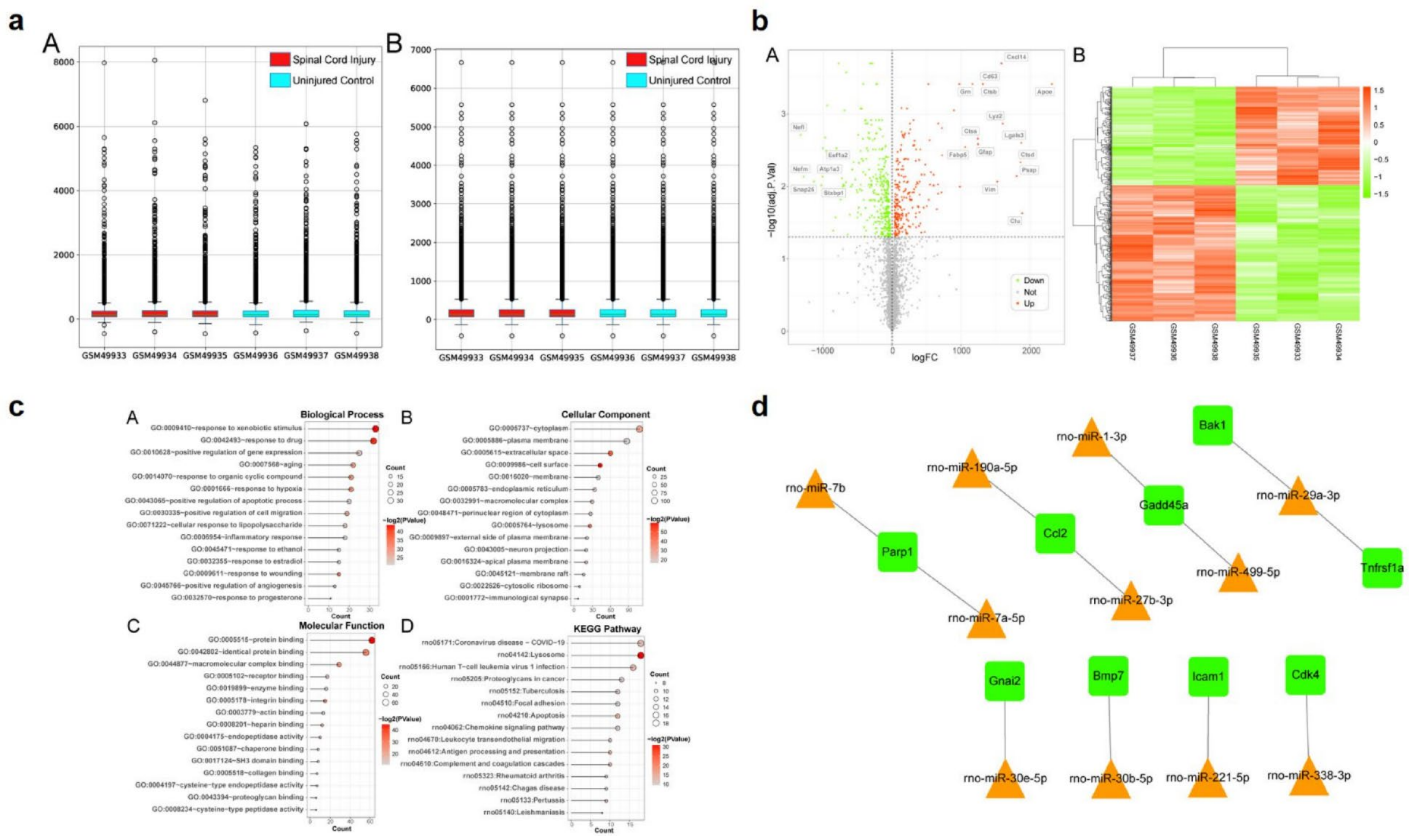
(**a**) Box plot (visualization of gene expression analysis). (**b**) Volcano plot (displaying results of differential expression analysis). (**c**) Bar plot (visualization of functional enrichment analysis results). (**d**) Network graph (showing nodes and interaction networks in the olecular regulatory network of the list)

After normalization of the GSE2599 dataset, the median levels and outliers in the boxplots were consistent (Fig. 1a). Using the limma package, we identified DEGs and labeled them with |logFC|> 1.0 (Fig. 1b). Hierarchical clustering analysis clearly distinguished between the SCI and normal control samples. A complete list of the upregulated and downregulated DEGs is provided in Table S3.

### Functional enrichment analysis of DEGs

GO and KEGG pathway analyses were performed using DAVID. Due to the large number of enriched terms, only the top 15 most significant terms (p-values) are shown (Fig. 1c). Upregulated DEGs were primarily enriched in biological processes related to oxidative stress, inflammatory responses, and apoptosis, whereas downregulated DEGs were mainly associated with neuronal conduction and synaptic functions.

### Gene-miRNA interaction analysis

From the KEGG pathways enriched in upregulated DEGs, we selected those related to oxidative stress, inflammatory responses, and apoptosis (Table 1). Using miRTarBase, we predicted the miRNAs targeting genes in these pathways (Fig. 1d), ultimately identifying 11 miRNAs and 9 target genes (Table 2). Through a comprehensive analysis, we selected miR499-5P for subsequent in vitro and in *vivo* studies.

**Table 1:** Pathways related to oxidative stress, inflammatory response and apoptosis

Term	Count	*P*-Value	Genes
rno04610:Complement and coagulation cascades	10	0.0000373	C1qb, Cfd, Itgam, Plau, Cr1l, C1r, Pros1, Plaur, Serping1, Clu
rno04612:Antigen processing and presentation	10	0.0000410	Cd74, Cd4, RT1-CE12, Ctsl, Psme1, Calr, B2m, Ctss, Ctsb, RT1-Da
rno04210:Apoptosis	12	0.0000582	Parp1, Ctsl, Gadd45a, Ctsk, Ctsh, Fos, Bak1, Ctsd, Ctss, Ctsc, Ctsb, Tnfrsf1a
rno05166:Human T-cell leukemia virus 1 infection	16	0.0000854	Nrp1, RT1-CE12, Tgfb1, Adcy4, Fos, Tgfbr1, Icam1, Tnfrsf1a, Cd4, Pttg1, Cdk4, Tspo, Calr, Tln1, B2m, RT1-Da
rno04670:Leukocyte transendothelial migration	10	0.0004030	Rap1b, Ocln, Itgam, Cldn7, Msn, Cxcr4, Cyba, Ezr, Gnai2, Icam1
rno04062:Chemokine signaling pathway	12	0.0007460	Cx3cr1, Lyn, Rap1b, Hck, Prkcd, Ccl3, Adcy4, Ccl2, Cxcr4, Cxcl14, Gnai2, Pf4
rno04064:NF-kappa B signaling pathway	8	0.0027258	Lyn, Parp1, Plau, Gadd45a, Lbp, Cd14, Icam1, Tnfrsf1a
rno04933:AGE-RAGE signaling pathway in diabetic complications	8	0.0032304	Col1a2, Tgfb1, Cdk4, Prkcd, Fn1, Ccl2, Tgfbr1, Icam1
rno04061:Viral protein interaction with cytokine and cytokine receptor	7	0.0057590	Cx3cr1, Ccl3, Ccl2, Cxcr4, Cxcl14, Pf4, Tnfrsf1a
rno04010:MAPK signaling pathway	12	0.0255850	Rap1b, Tgfb1, Gadd45a, Mapkapk2, Hspb1, Fos, Flnc, Cd14, Tgfbr1, Pgf, Dusp7, Tnfrsf1a
rno04620:Toll-like receptor signaling pathway	6	0.033119975	Ctsk, Ccl3, Spp1, Fos, Lbp, Cd14
rno04060:Cytokine-cytokine receptor interaction	11	0.034456052	Cx3cr1, Cd4, Tgfb1, Ccl3, Ccl2, Cxcr4, Cxcl14, Bmp7, Tgfbr1, Pf4, Tnfrsf1a
rno04666:Fc gamma R-mediated phagocytosis	6	0.035827928	Lyn, Hck, Arpc1b, Inpp5d, Prkcd, Fcgr2b

**Table 2:** 11 miRNAs and 9 target genes

Gene	Pathway
Bak1	Apoptosis
Bmp7	Cytokine-cytokine receptor interaction
Ccl2	Chemokine signaling pathway, AGE-RAGE signaling pathway in diabetic complications, Viral protein interaction with cytokine and cytokine receptor, Cytokine-cytokine receptor interaction
Cdk4	Human T-cell leukemia virus 1 infection, AGE-RAGE signaling pathway in diabetic complications
Gadd45a	Apoptosis, NF-kappa B signaling pathway, MAPK signaling pathway
Gnai2	Leukocyte transendothelial migration, Chemokine signaling pathway
Icam1	Human T-cell leukemia virus 1 infection, Leukocyte transendothelial migration, NF-kappa B signaling pathway, AGE-RAGE signaling pathway in diabetic complications
Parp1	Apoptosis, NF-kappa B signaling pathway
Tnfrsf1a	Apoptosis, Human T-cell leukemia virus 1 infection, NF-kappa B signaling pathway, Viral protein interaction with cytokine and cytokine receptor, MAPK signaling pathway, Cytokine-cytokine receptor interaction

## MSC-EXO characterization and miRNA screening

**Fig. 2:**
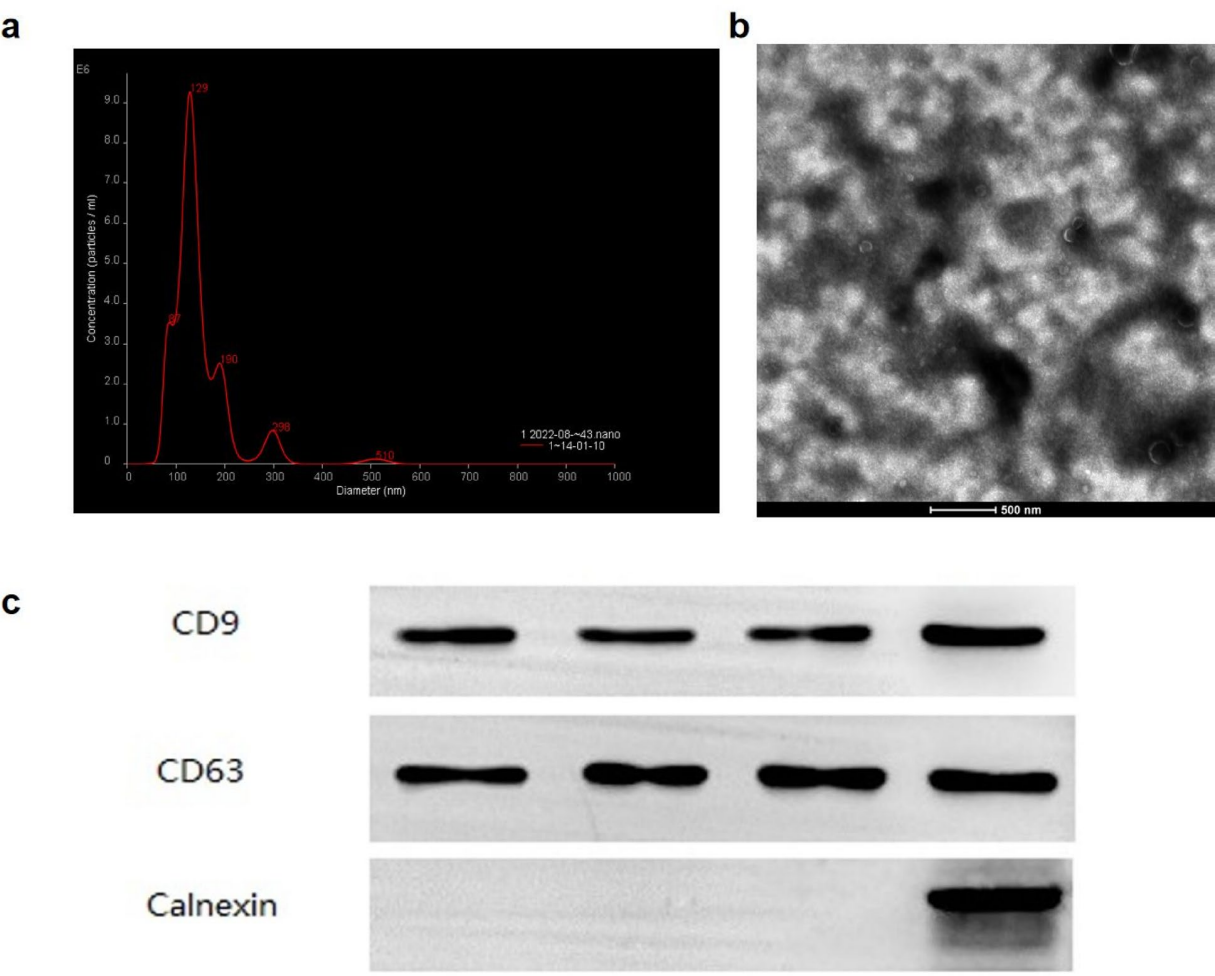
Isolation, characterization, and uptake assay of MSC-EXO. (**a**) Nanoparticle tracking analysis (NTA) of exosomes. (**b**) Electron microscopy imaging of exosomes. (**c**) Western blotting. (WB) results of exosome-specific marker proteins

NTA revealed that MSC-EXO had an average size of 145.3 nm, with a primary peak at 104.9 nm, and 89.9% of exosomes were within the 30–200 nm range (Fig. 2a). Transmission electron microscopy showed a typical cup-shaped morphology (Fig. 2b). Western blotting analysis confirmed the positive expression of the exosomal markers CD9 and CD63 (Fig. 2c), verifying successful exosome isolation.

**Fig. 3:**
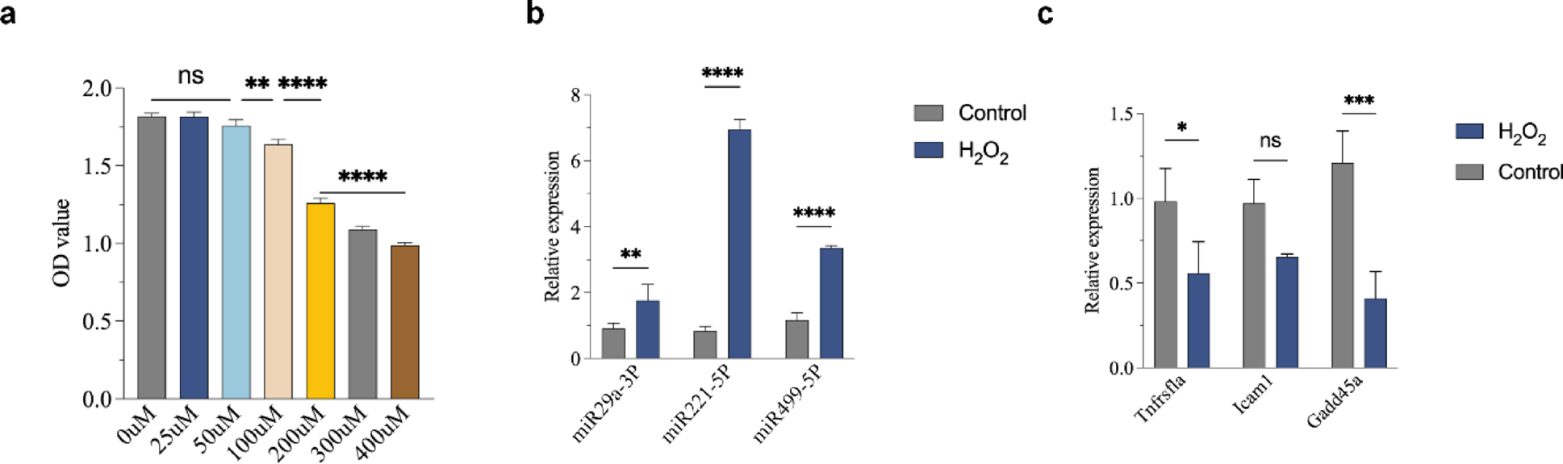
Screening for optimal miRNAs to construct engineered MSC-EXO. (**a**) CCK-8 assay to determine H₂O₂ concentration and treatment duration. (**b**) qPCR analysis of the effects of H₂O₂ treatment on miRNAs and their target genes. (**c**) Luciferase reporter gene assay. (n = 3 per group), Error bars indicate the SD (*P < 0.05, **P < 0.01, ***P < 0.001, ****p < 0.0001)

To select the optimal miRNAs for engineered MSC-EXO, we evaluated the effects of different miRNAs on PC12 cells under oxidative stress. CCK-8 assays showed that treatment with 200 μM H_2_O_2_ for 12 h significantly reduced PC12 cell viability while maintaining sufficient cell survival in subsequent experiments (Fig. 3a). RT-qPCR analysis demonstrated that among miR29a-3P, miR221-5P, and miR499-5P, miR499-5P had the most significant regulatory effect on its target genes under oxidative stress (Fig. 3b). Luciferase reporter assays further confirmed that miR499-5P directly bound to Gadd45a and induces translational repression/mRNA degradation (Fig. 3c). Based on these results, we selected miR499-5P to construct the engineered MSC-EXO-miR499-5P for subsequent studies.

### Engineered MSC-EXO-miR499-5P promotes PC12 cell growth

**Fig. 4:**
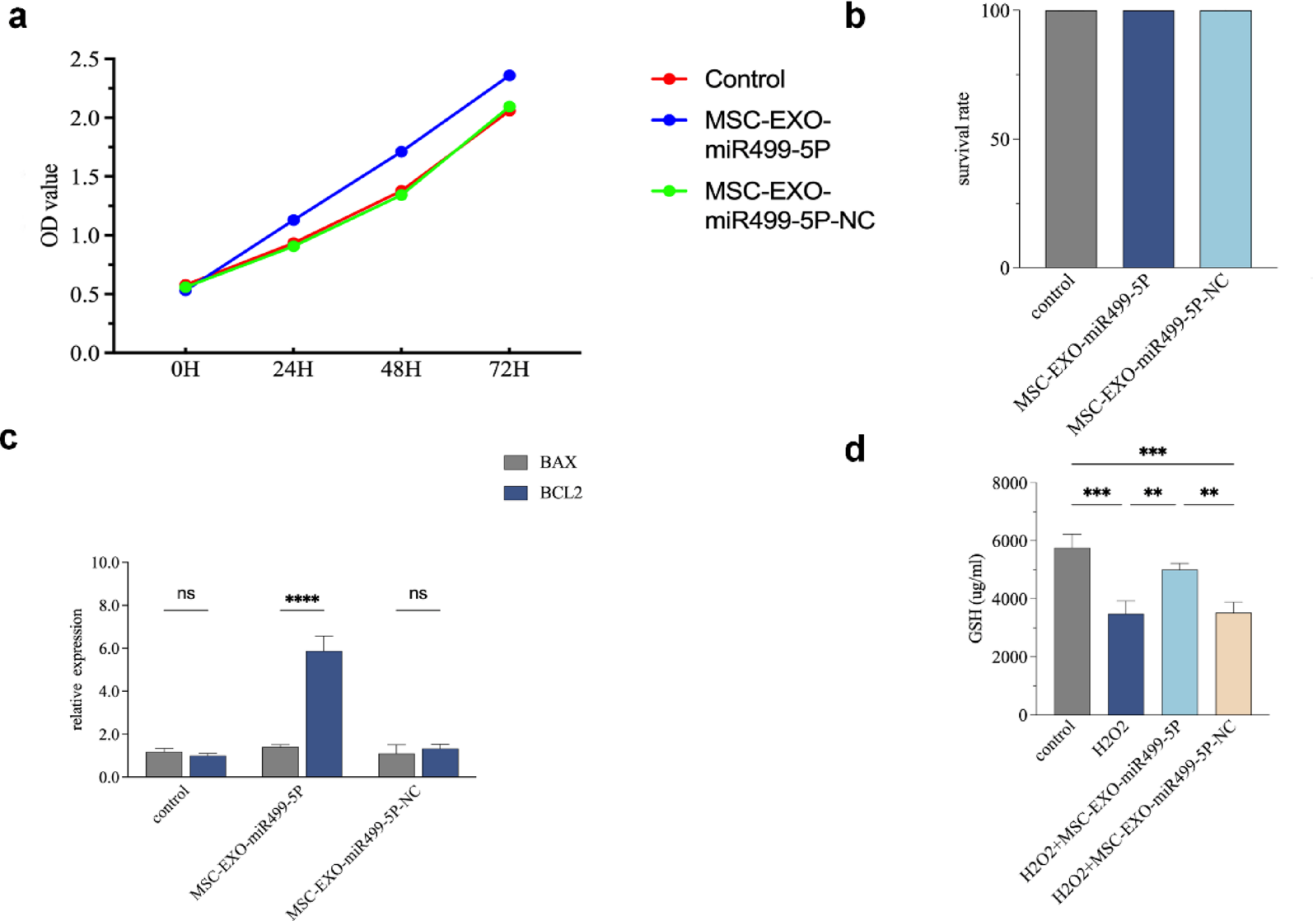
Engineered MSC-EXO-miR499-5P promotes PC12 cell growth. (**a**) CCK-8 assay to validate its effect on PC12 cell proliferation. (**b**) Flow cytometry results. (**c**) RT-qPCR results. (**d**) Western blotting validation results. (n = 3 per group), Error bars indicate the SD (*P < 0.05, **P < 0.01, ***P < 0.001, ****p < 0.0001)

The CCK-8 assay showed that MSC-EXO-miR499-5P significantly promoted the proliferation of PC12 cells (Fig. 4a). Flow cytometric analysis revealed that MSC-EXO-miR499-5P did not induce apoptosis in PC12 cells (Fig. 4b). Furthermore, RT-qPCR and western blot analyses demonstrated that MSC-EXO-miR499-5P upregulated Bcl2 transcription and protein expression, thereby inhibiting apoptosis (Fig. 4c, d). These results indicated that MSC-EXO-miR499-5P promoted PC12 cell growth by enhancing proliferation and suppressing apoptosis.

### Engineered MSC-EXO-miR499-5P inhibits oxidative stress and inflammatory response in injured PC12 cells

**Fig. 5:**
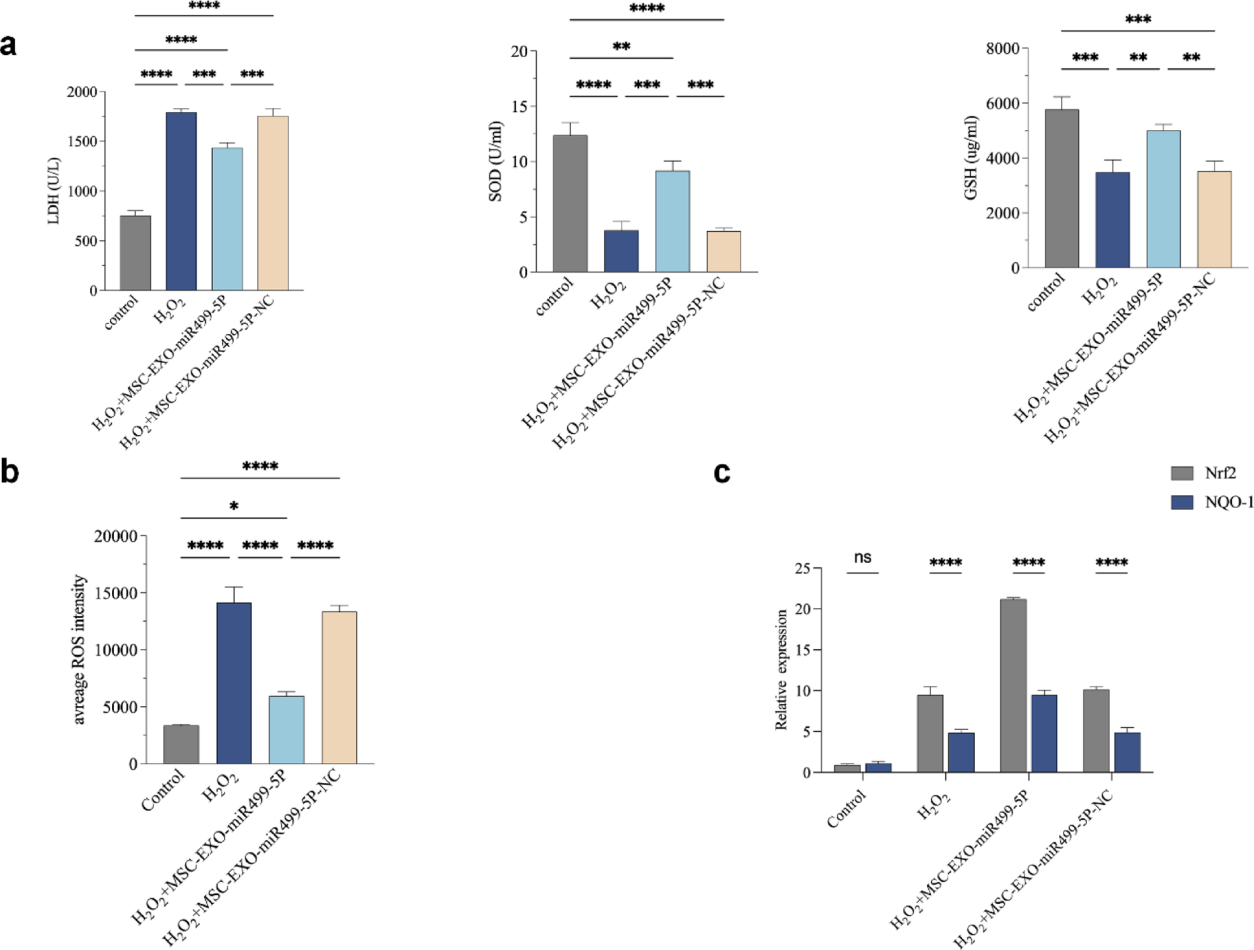
Engineered MSC-EXO-miR499-5P suppresses oxidative stress and inflammatory response in injured PC12 cells. (**a**) LDH, SOD, and GSH levels: After co-culture with MSC-EXO-miR499-5P, extracellular LDH decreased, while SOD and GSH levels were higher than in the model group. (**b**) Flow cytometry analysis of DCFH-DA probe loading in differently treated PC12 cells. (**c**) RT-qPCR results (H₂O₂-treated cells showed increased expression of stress-related genes Nrf2 and NQO1; MSC-EXO-miR499-5P further enhanced their expression, improving oxidative stress resistance). (n = 3 per group), Error bars indicate the SD (*P < 0.05, **P < 0.01, ***P < 0.001, ****p < 0.0001)

Biochemical assays showed that H_2_O_2_ treatment significantly increased extracellular LDH release (an indicator of cell damage) and altered SOD and GSH levels (indicative of oxidative stress) in PC12 cells (Fig. 5a). MSC-EXO-miR499-5P treatment effectively reversed these effects. Flow cytometric analysis using the DCFH-DA probe confirmed that MSC-EXO-miR499-5P significantly reduced intracellular reactive oxygen species (ROS) levels (Fig. 5b). RT-qPCR and western blot analyses further demonstrated that MSC-EXO-miR499-5P enhanced the expression of the antioxidant factors NRF2 and NQO-1 (Fig. 5c).

**Fig. 6:**
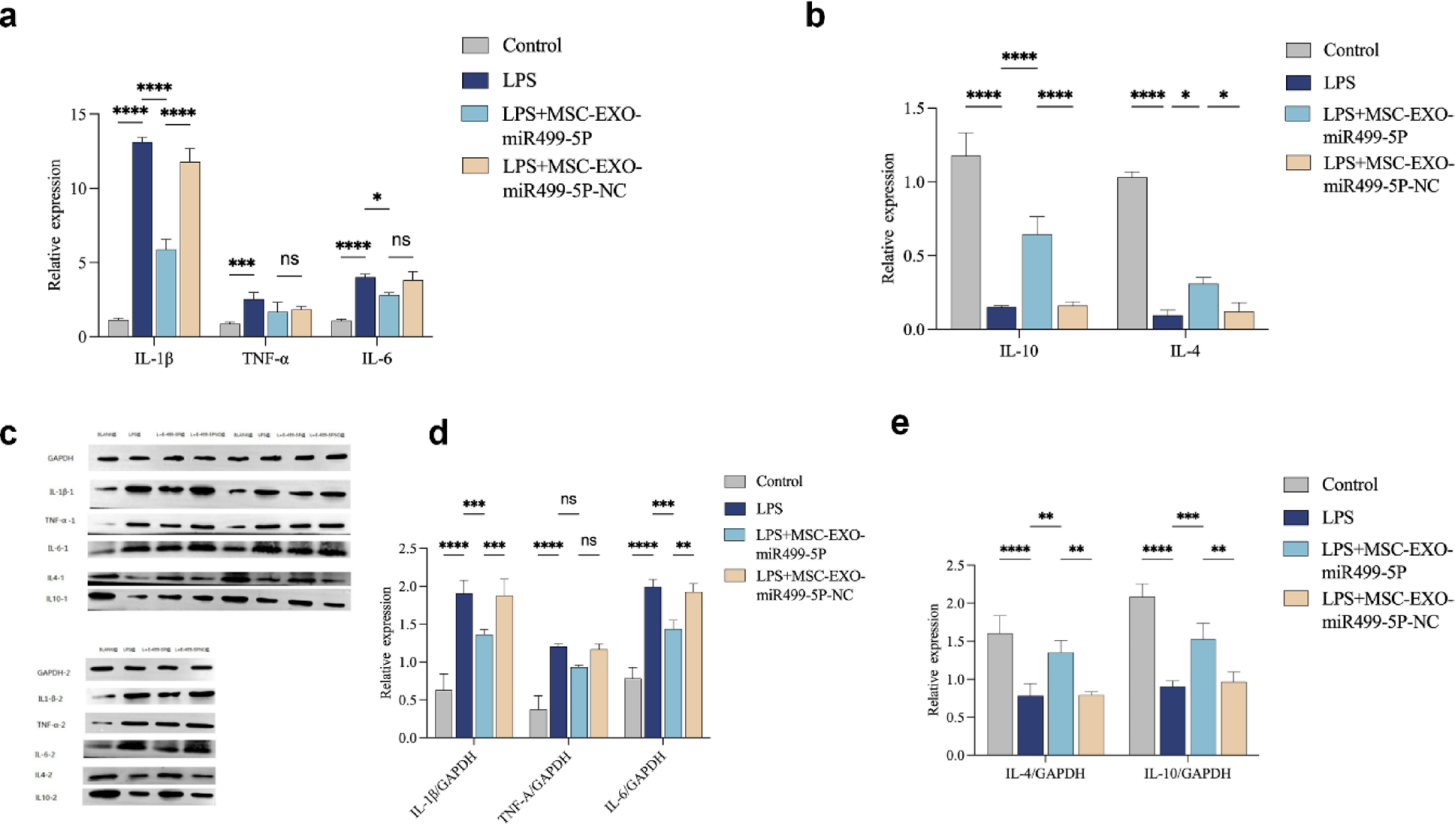
qPCR and WB analysis of pro-/anti-inflammatory gene expression in LPS-stimulated PC12 cells. (**a**) Expression of IL-1β, TNF-α, and IL-6 genes in differently treated PC12 cells. (**b**) Expression of IL-10 and IL-4 genes in differently treated PC12 cells. (**c**) Western blotting results. (n = 3 per group), Error bars indicate the SD (*P < 0.05, **P < 0.01, ***P < 0.001, ****p < 0.0001)

In the inflammatory model, LPS stimulation significantly increased the expression of pro-inflammatory cytokines (IL-1β, TNF-α, and IL-6) and decreased the expression of anti-inflammatory cytokines (IL-10 and IL-4) in PC12 cells (Fig. 6). MSC-EXO-miR499-5P treatment effectively reversed these changes, demonstrating its potent anti-inflammatory effect.

### Engineered MSC-EXO-miR499-5P repairs spinal cord microenvironment and promotes functional recovery after SCI

**Fig. 7:**
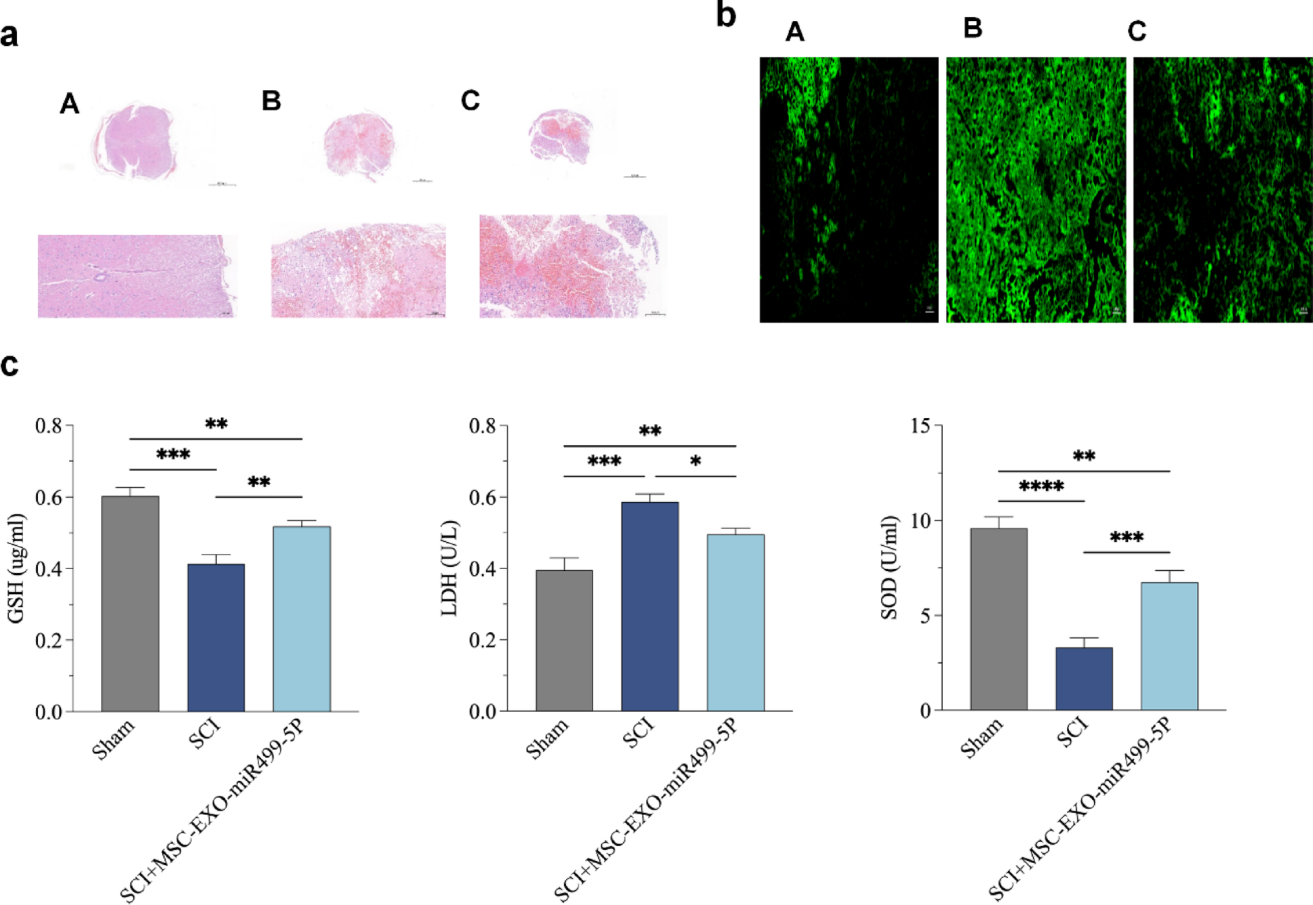
Engineered MSC-EXO-miR499-5P repairs the spinal cord microenvironment in SCI mice. (**a**) HE staining of spinal cord pathological changes (A: Control group; B: Model group; C: Treatment group). (**b**) ROS detection of reactive oxygen species (ROS) changes in the spinal cord (A: Control group; B: Model group; C: Treatment group) (After spinal cord injury, ROS levels significantly increased; post-treatment, ROS decreased but remained higher than in the sham group). (**c**) Biochemical analysis of LDH, GSH, and SOD levels in spinal cord tissue (In the model group, SOD and GSH decreased while LDH increased after injury; in the treatment group, SOD and GSH increased while LDH decreased). (n = 3 per group), Error bars indicate the SD (*P < 0.05, **P < 0.01, ***P < 0.001, ****p < 0.0001)

**Fig. 8:**
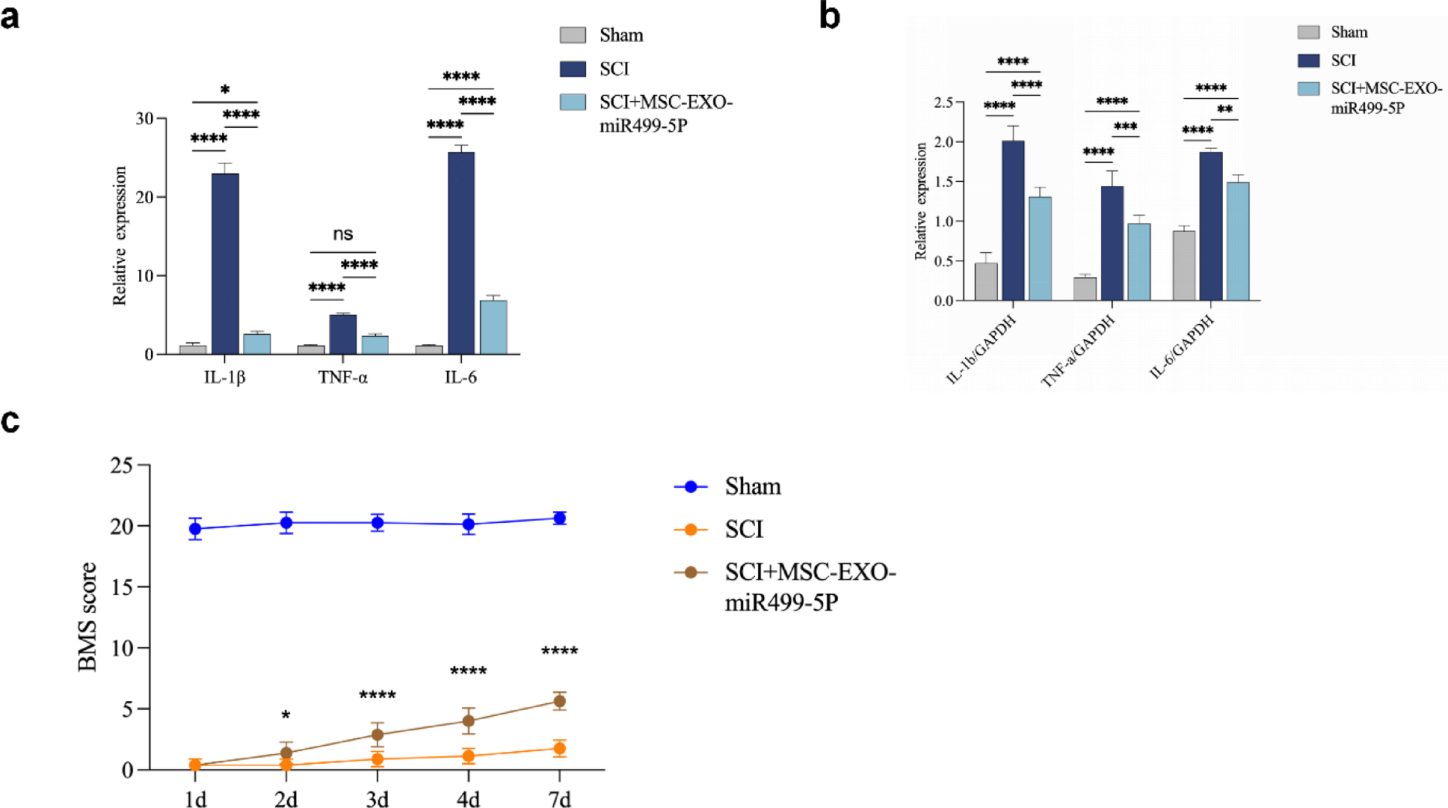
(**a**) qPCR analysis of pro-/anti-inflammatory gene expression in mouse spinal cord. (**b**) WB detection of pro-/anti-inflamm atory protein expression in mouse spinal cord. (**c**) BBB score. (n = 3 per group), Error bars indicate the SD (*P < 0.05, **P < 0.01, ***P < 0.001, ****p < 0.0001)

Histopathological examination revealed severe hemorrhage, necrosis, and microglial infiltration in the SCI group, with loss of gray-white matter boundaries (Fig. 7a). MSC-EXO-miR499-5P treatment significantly improved these pathological changes. Biochemical analysis showed that MSC-EXO-miR499-5P treatment normalized the levels of oxidative stress markers (including ROS, LDH, GSH, and SOD) in spinal cord tissue (Figs. 7b, c). The treatment also effectively regulated the expression of inflammatory cytokines, reduced pro-inflammatory factors (IL-1β, TNF-α, and IL-6), and increased anti-inflammatory factors (IL-10 and IL-4) (Fig. 8a, b). BMS scoring demonstrated a significant improvement in motor function recovery in the treatment group (Fig. 8c).

Collectively, these results demonstrated that MSC-EXO-miR499-5P can effectively repair the spinal cord microenvironment after SCI by modulating oxidative stress, inflammation, and apoptosis, thus promoting functional recovery.

## Discussion

Spinal cord injury (SCI) is a devastating condition with high morbidity and mortality rates. Catastrophic injuries frequently lead to severe motor dysfunction below the injury level, and even death. Despite extensive research in the past few decades, effective treatments for SCI remain limited [53–55].

Recent studies have highlighted the crucial roles of miRNAs in the regulation of exosomal functions. Increasing evidence suggests that exosome-transported miRNAs from various cellular sources exhibit remarkable protective effects against SCI. For instance, a clinical study confirmed that miR199a-3P/145-5P, which are highly expressed in exosomes, promote the differentiation of LPS-inhibited PC12 cells in vitro by modulating the NGF/TrkA pathway. Furthermore, these exosomal miRNAs were found to target Cblb and Cbl genes, influencing TrkA ubiquitination and activating the NGF/TrkA pathway, ultimately promoting axonal development and motor function recovery in rats with SCI [56]. Another study revealed that neuron-derived exosomes enhanced functional recovery after SCI by suppressing the activation of M1 microglia and A1 astrocytes both in vivo and in vitro. Using a miRNA array analysis, miR124-3P was identified as the most abundant miRNA in neuron-derived exosomes, with MYH9 as its downstream target gene. Subsequent experiments confirmed the miR124-3P/MYH9 axis and suggested the potential involvement of the PI3K/AKT/NF-κB signaling cascade in exosomal miR124-3P-mediated microglial regulation [57]. Collectively, these findings suggest that miRNAs, particularly those derived from exosomes, hold great promise as effective therapeutic agents for neural recovery after SCI, highlighting the importance of exploring novel miRNAs for SCI treatment.

For bioinformatic analysis, we downloaded the GSE2599 dataset from the GEO database. To identify DEGs between h SCI and healthy controls, we employed the limma package to analyze gene expression differences, with p-values adjusted using the FDR method. Using the miRTarBase database, we predicted miRNA-target interactions by focusing on genes related to oxidative stress, inflammatory responses, and apoptosis. Through comprehensive comparison and analysis, we selected miR499-5P for subsequent in vitro and in vivo experiments.

In our in vitro experiments, CCK-8 assays demonstrated that engineered MSC-EXO-miR499-5P significantly enhanced PC12 cell proliferation. Flow cytometry, RT-qPCR, and western blotting analyses further confirmed that MSC-EXO-miR499-5P promoted PC12 cell growth by stimulating proliferation and suppressing apoptosis. Co-culture experiments revealed that MSC-EXO-miR499-5P treatment reduced extracellular LDH levels and increased SOD and GSH activities compared to those in the H_2_O_2_-treated group, indicating attenuated cellular damage and oxidative stress. Additionally, the engineered exosomes markedly decreased the expression of pro-inflammatory cytokines (IL-1β, TNF-α, and IL-6) while upregulating anti-inflammatory factors (IL-10 and IL-4), demonstrating potent anti-inflammatory effects.

In vivo studies showed that MSC-EXO-miR499-5P treatment significantly improved the pathological features of SCI in mice. Histopathological analysis revealed reduced hemorrhage, decreased microglial infiltration, suppressed inflammatory responses, and reduced neuronal necrosis in treated animals. Biochemical assays demonstrated significantly lower ROS and LDH levels, along with elevated GSH content and SOD activity in the spinal cord tissues of the treatment group. Moreover, the expression of pro-inflammatory cytokines (IL-1β, TNF-α, and IL-6) was substantially downregulated, whereas that of anti-inflammatory cytokines (IL-10 and IL-4) was upregulated in the treated mice. Most importantly, BMS scoring confirmed that MSC-EXO-miR499-5P treatment significantly promoted motor function recovery in mice with SCI.

A notable limitation of this study is the absence of an MSC-EXO-only control group in vivo. While our in vitro findings demonstrate the therapeutic potential of MSC-EXO-miR499-5P in promoting neural recovery after spinal cord injury, we acknowledge that not including this control group in vivo limits our ability to fully distinguish between the effects of MSC-EXO alone and the specific contribution of miR499-5P. The inclusion of this control group in vivo could provide a more comprehensive understanding of how MSC-EXO and miR499-5P independently contribute to the observed therapeutic outcomes. However, due to the scope of this study, which primarily aimed to investigate the role of miR499-5P within MSC-EXO, and considering the additional resources and time required for this experiment, we did not include the MSC-EXO-only control group in vivo. This limitation should be taken into account when interpreting the results, and future studies may address this by incorporating a more extensive experimental design. In addition, we chose to administer 50 μL of MSC-EXO-miR499-5P solution daily for seven consecutive days (a total of 350 μL). This dosage may potentially lead to local edema or compression. During the experiment, we closely monitored the local reactions of the mice and found no significant edema or tissue compression. However, due to the relatively large injection volume, there is still a potential possibility of some impact on the local tissue of the mice. In addition, this study used chemically synthesized miRNA mimics, which are derived from rat miRNA, but the experimental model is based on mice. Due to interspecies differences, although rats and mice share many biological similarities, the use of miRNA mimics across species may affect the targeting effects of the miRNA, potentially influencing the experimental results.

## Conclusion

In conclusion, our engineered MSC-EXO-miR499-5P effectively promoted cell growth, alleviated oxidative stress and inflammatory responses following cellular injury, and contributed to repair of the spinal cord microenvironment while enhancing functional recovery after SCI. As the first systematic investigation of miR499-5P's therapeutic potential for SCI, this study demonstrates its promise for future clinical applications. Therefore, engineered MSC-EXO-miR499-5P represents a novel and potentially effective therapeutic strategy for spinal cord injury treatment.

## Supplementary Information

Below is the link to the electronic supplementary material.

Supplementary Material 1

## Data Availability

Data supporting the findings of this study are publicly available in the GEO database under accession number GSE2599 (https://www.ncbi.nlm.nih.gov/geo/query/acc.cgi?acc=GSE2599) and miRTarBase database (http://mirtarbase.cuhk.edu.cn/).
