# Supplementary material for: MiR499-5P loaded MSC derived exosomes affect oxidative stress and inflammatory response after spinal cord injury by targeting genes
Source: J Orthop Surg Res. 2025 Dec 10;20:1068. doi: 10.1186/s13018-025-06500-w (PMC12696906; doi:10.1186/s13018-025-06500-w)
Supplement: Supplementary file 1 — Supplementary Material 1 [file 13018_2025_6500_MOESM1_ESM.docx]

**Table S1.** The complete lists of up-regulated and down-regulated DEGs.

| Up regulated DEG | Down regulated DEG |
| --- | --- |
| Genes | Genes |
| Cxcl14 | Cyp51 |
| Grn | Hmgcs1 |
| Ctsb | Acat2 |
| Cd53 | Msmo1 |
| C1qb | Cyfip2 |
| Cd63 | Ndufb8 |
| Apoe | Cadps |
| Cd37 | Uchl1 |
| P2rx4 | Vamp1 |
| Hspb1 | Fdps |
| Arl11 | Nefh |
| LOC360231 | Sncb |
| Lyz2 | Psmc1 |
| Vat1 | Scg5 |
| Ifitm3 | Fdft1 |
| Fcgr2b | Ppp2r2b |
| Ppap2c | Dock9 |
| Rab13 | Sqle |
| Cp | Tuba4a |
| Apobec1 | Pcp4 |
| Esrra | Dync1i1 |
| Hmox1 | Cabp1 |
| Plin2 | B3galt5 |
| Ctss | Penk |
| Txnip | Ppp3ca |
| Sgk1 | Slc25a1 |
| Lgals3 | Scd1 |
| Gfap | Nefl |
| Fos | Vsnl1 |
| Gabarap | Ndn |
| Tln1 | Iqsec1 |
| Fabp5 | Thy1 |
| Timp1 | Fbxo2 |
| Ezr | Gad1 |
| Srebf1 | Cplx1 |
| Itpkb | Insig1 |
| Rgs1 | Ptpn5 |
| Timp2 | Cdk18 |
| Arsb | Ndrg4 |
| Hk2 | Eef1a2 |
| Entpd2 | Ngfrap1 |
| Bhlhe41 | Ache |
| Prkcd | Klhdc3 |
| Rpl18 | Calb2 |
| Tpm3 | Pcp4l1 |
| Gnai2 | Aes |
| Cldn7 | Prkar1b |
| Ctsd | ND4 |
| Dock8 | Sst |
| Tep1 | Srd5a1 |
| Pttg1ip | RGD1311739 |
| Cbfb | Trim3 |
| Slc6a8 | Mgst3 |
| Cmtm6 | Cacna2d1 |
| Hck | Mapk8ip2 |
| Casp1 | Ptplad1 |
| Mgst1 | Gnai1 |
| Gusb | Mdh1 |
| Pdpn | Pc |
| Cyba | Bcat1 |
| Anxa2 | Ndufa4 |
| Pros1 | Uqcc2 |
| Ctsc | Kcnc1 |
| Psap | Dut |
| Litaf | Gls |
| Pdlim4 | Cck |
| Ctse | Mog |
| Fcgrt | Nsg1 |
| Ucp2 | Snf8 |
| Vim | Ncdn |
| RT1-Da | Mobp |
| Plscr3 | Olfm1 |
| Tspo | Got1 |
| Pmp22 | Lin7b |
| Arpc1b | Rab3a |
| Adcy4 | Syn1 |
| RT1-CE12 | Pkia |
| Ptn | Slc32a1 |
| LOC100912677 | Gabbr2 |
| Rpl22 | Nefm |
| Adam17 | Prkaca |
| Atf3 | Atp1a3 |
| Gpx1 | Lysmd4 |
| Rnf4 | Cox5b |
| Tgfbr1 | Camkk1 |
| S100a4 | Idi1 |
| Ctsh | Ina |
| Ccl3 | Pea15 |
| Soat1 | Rab6a |
| Aldh2 | Amigo1 |
| Lcat | Chn1 |
| Gadd45a | Prune2 |
| Tgfb1 | Rtn1 |
| Scn7a | Zmat3 |
| Fuca1 | Agtpbp1 |
| Psme1 | Tango2 |
| Rap1b | Oxr1 |
| RT1-Cl | Pvalb |
| Anxa1 | Snap25 |
| Mmp14 | Gucy1b3 |
| Gns | Mfsd6 |
| Btg1 | Pde2a |
| Itgam | Pdhb |
| Rps5 | Tceb2 |
| Mmp12 | Rapgef4 |
| Col5a2 | Csdc2 |
| Gch1 | Grik4 |
| Tpm4 | Syp |
| Lxn | Cnr1 |
| Ctsk | Cntnap1 |
| Scamp2 | Cdk16 |
| Cxcr4 | Nrgn |
| Lyn | Mapt |
| Sh2b2 | Stx1b |
| Calr | Pebp1 |
| Col1a2 | Akr7a3 |
| Cnn3 | Nrn1 |
| Tmed2 | Rasgrf1 |
| Slc16a1 | Ckmt1b |
| Tmem53 | Apbb1 |
| Plau | Stxbp1 |
| Renbp | Epb4.1 |
| P4hb | Map6 |
| Mdfic | Hsph1 |
| Slc15a4 | Dnm1 |
| Ccl2 | Cnih2 |
| Id1 | Ndrg3 |
| Bak1 | Ywhah |
| Icam1 | Rnf112 |
| Ocln | Ywhag |
| Gbp2 | Uqcrfs1 |
| Cux1 | Pdzd4 |
| Serping1 | Fkbp2 |
| LOC102555086 | Kcnk1 |
| Id3 | Auh |
| Pf4 | Tacr1 |
| Ptpn1 | Rab4a |
| Calcrl | Ank3 |
| Dmgdh | Pcyt2 |
| Dab2 | Endod1 |
| Aqp1 | Tnk2 |
| Crip2 | Pip5k1c |
| Rpl3 | Eno2 |
| Coro1a | Tmem126b |
| Aebp1 | Scn1a |
| B2m | Tgfa |
| Cx3cr1 | Slc12a5 |
| Oplah | Hpcal1 |
| Mapkapk2 | Gls2 |
| Spp1 | Fgf9 |
| Arhgap25 | Dlg4 |
| Gne | Ivns1abp |
| Slfn2 | Syn2 |
| Parp1 | Pdha1 |
| Rpl14 | Sptbn2 |
| Cr1l | Atp5c1 |
| Prkab1 | Prkacb |
| Kpnb1 | Atp5d |
| Clu | Acsl3 |
| Ctsl | Rabgap1l |
| Dgkg | Trim32 |
| Ninj1 | Guk1 |
| Aqr | Hiat1 |
| Reln | Sds |
| Erp29 | Slc2a1 |
| Bmp7 | Paip1 |
| Slc5a3 | Limk1 |
| Nrp1 | Atp5j |
| Ptpro | Chga |
| Tnfrsf1a | Zfp180 |
| Rpl36 | Cox6a1 |
| Slc15a2 | Asic2 |
| Plaur | Atp9a |
| Lamc1 | Ndufa8 |
| Mcm6 | Enpp2 |
| Cstb | Pfdn2 |
| Msn | Nolc1 |
| Clec11a | Fn3k |
| Cfd | Abi2 |
| Ppif | Faim2 |
| Eaf1 | Kcnab2 |
| Gpx2 | RGD1306941 |
| Cdk4 | Mtmr7 |
| Plod1 | Tomm6 |
| Dcakd | Cx3cl1 |
| Sin3b | Map1a |
| LOC102555617 | RGD1309779 |
| Inpp5d | Ciao1 |
| Bgn | Myo1a |
| Cd4 | Cds1 |
| Tbc1d2b | Dtd1 |
| Flnc | Ppp3r1 |
| Cav1 | Matk |
| Slc2a5 | Gjb1 |
| Pttg1 | Pde6d |
| Dusp7 | B4galnt1 |
| Ptger2 | Rictor |
| Isl2 | Pgam1 |
| Eef1a1 | Rab5a |
| Arl4a | Ldhb |
| B4galt1 | Sh3gl2 |
| Cd74 | Gskip |
| Pfn1 | Dnaja3 |
| Mcrs1 | Slc4a3 |
| Acsl5 | Me1 |
| Rcn3 | Stip1 |
| Ptpn6 | Nptn |
| Wdr31 | Sh3gl3 |
| Itgb4 | Rnf157 |
| Lgals3bp | Tac3 |
| Ece1 | Inadl |
| Fn1 | Fez1 |
| Fzd1 | Gnb5 |
| Npr1 | Gabrg2 |
| Ech1 | Pnck |
| Zfp36l1 | Gnpat |
| Ndst1 | Gtf2a1 |
| Irf1 | Ncs1 |
| Lbp | Pex14 |
| Man2b1 | Dpp6 |
| Tnxa-ps1 | Sigmar1 |
| Itga1 | Inpp4a |
| Rpl24 | Pak1 |
| Srpx | Tmeff1 |
| Ptprf | Mvd |
| Akr1a1 | Chchd6 |
| Vhl | Vmp1 |
| Fabp7 | Ddt |
| Lipa | Cdc42bpb |
| Rhoc | Cox4i1 |
| Rps3a | Elavl4 |
| Cnpy2 | Aldoa |
| C1r | Slc25a3 |
| Aco1 | Hmox2 |
| Acp5 | Hpca |
| Aprt | Clcn4 |
| Smarcd2 | Nme1 |
| Copz1 | Tnnc2 |
| Pgf | Dhcr7 |
| Gap43 | Tprg1l |
| Cd14 | Psmd1 |
| Psmb9 | Srpk2 |
| Rps24 | Svop |
|  | Mapk6 |
|  | Qdpr |
|  | Ndufa3 |
|  | LOC100362572 |
|  | Spock2 |
|  | Psmc5 |
|  | Nell2 |
|  | Atp5a1 |
|  | Comt |
|  | Gabbr1 |
|  | Tnfrsf11b |
|  | Pkp4 |
|  | Hmgcr |
|  | Chgb |
|  | Chn2 |
|  | Kcns3 |
|  | Scn1b |
|  | Mapk8ip1 |
|  | Polr3k |
|  | Ppt1 |
|  | Gnal |
|  | Sepw1 |
|  | Lss |
|  | Aktip |
|  | H2afx |
|  | Cdk5 |
|  | Nrxn3 |
|  | Cox7b |
|  | Tac1 |
|  | Begain |
|  | Rhbdl1 |
|  | Tsc2 |
|  | Plcb1 |
|  | Nsf |
|  | Kif3c |
|  | Kcnt1 |
|  | Mif |
|  | Ero1lb |
|  | Prdx3 |
|  | Leprel4 |
|  | Ppm1a |
|  | Grm1 |
|  | Scamp1 |
|  | Pde1b |
|  | P2rx5 |
|  | Tagln3 |
|  | Mark1 |
|  | Acsl6 |
|  | Atp2a2 |
|  | Arhgef9 |
|  | Gsk3a |
|  | Dcaf6 |
|  | Cadps2 |
|  | Hagh |
|  | Ndufb10 |
|  | Thrsp |
|  | Akap12 |
|  | Atp2b3 |
|  | Evl |
|  | Cipc |
|  | Pnpo |
|  | ND2 |
|  | Gpx4 |
|  | RGD1305587 |
|  | Slc18a3 |
|  | Idh3g |
|  | Sv2a |
|  | Camk2n2 |
|  | Enpp6 |
|  | Cnrip1 |
|  | Mdh2 |
|  | Papss1 |
|  | Pfkm |
|  | Acvr2a |
|  | Ndufb5 |
|  | Schip1 |
|  | Abcb1a |
|  | Ebp |
|  | Gnas |
|  | Slc8a3 |
|  | Pygb |
|  | Rims1 |
|  | Tpd52 |
|  | Frmd5 |
|  | Clip2 |
|  | Acot7 |
|  | Pop1 |
|  | Tspan3 |
|  | Nap1l4 |
|  | Pex19 |
|  | Atg13 |
